# On the Charges of Country Practitioners for Occasional Visits to Paupers, in Answer to Humanitas, in the Journal for August

**Published:** 1817-10

**Authors:** 


*For the London Medical and Physical Journal.*

*On the Charges of Country Practitioners for occasional Visits to Paupers, in Answer to Humanitas, in the Journal for August;* by VIGORNIENSIS.

HUMANITAS, in the heat of his feelings, has suffered his judgment to be carried away. I shall endeavour to shew that the proposal of the Worcestershire Medical and Surgical Society has for its object the bettering the condition of the poor in regard to medical aid, in shewing of which it will be agreed they are adding to the respectability of the profession, and the charge of insult must fall to the ground.

First of all, sir, as to the insult offered to the profession at large. The foundation of such an application to Parliament has for its object the bettering of the condition of the poor, inasmuch as a service likely to be remunerated, as a general principle, begets more attention than when the only reward (consciousness of service) is the sole stimulus. By this it will be seen that, where duty and good to our fellow creature is a superior impulse, I take it as a deviation, but which, being found among so many of the profession, may well be mistaken for a much more extensive class, or even for a principle. The commerce of the world teaches there are two sets of practitioners,—one that no pecuniary stimulus will alone stir, the other that will not stir without. Which is the larger portion, every man may settle as he thinks best: it is only sufficient for my purpose to shew the poor are in the hands of both. Now, if the poor were alone in the hands of those who only want the stimulus of a worthy action, then there might be a greater plea and foundation for a charge of insult. But it is not alone the man in good practice we are to legislate for, but it is the professional man who is struggling with difficulties, who is able, willing to offer a thousand acts of attention, of kindness—who does it by manner, by feeling, yet is able to offer little else. Should the poor man fall into such hands, his situation is bettered, because the power of benevolence has been extended by a slight remuneration, a part of which the poor man will partake, for it is the very nature of such a character to expand. Look at it in the interested man's hands,—you apply to him his only stimulus; then surely, in either case, the poor are benefited. Even in the hands of the man in extended practice, it enlarges his sphere of charity.

Though it may be questioned by some whether my opinions may be correct, yet I should think there could be no dispute of the hard operation of the poor-laws, as they now stand, as they regard numerous manufacturers, who have found employment far from home. When overtaken by sickness they apply not to the parish for relief, for they well know, when their own parish are to be put to expence, they are removed. The man pines in sickness, for his situation does not become known: he is unwilling to put himself to the expence of medical attendance, or, perhaps, unable. In asking a remedy for such an occurrence, are we consulting the poor's benefit, insulting the profession, or endeavouring to put such an event out of the pale of accident? or are we seeking to provide medical attendance for every one on its surest basis—interest?

As to the quantum of remuneration, that is left perfectly to the wisdom of Parliament. A public remuneration never can be commensurate to the duties of such an office; but there is no reason why some should not be afforded,—and any means that can contribute to the better attendance on the poor maintains, instead of lessens, our dignity. No man of any respectability ever refuses his aid: I can feel my own duty, and I should never have questioned that of Humanitas, had he left out his gratuitous services—they are not uncommon—we ourselves may be silent; the poor will record them.

But on what principle we are to be more studious of the poor-rates than the butcher, the baker, or any other dealer, I am at a loss to guess. Our commodities are purchased by time, by expenditure, anxiety, by constant exertion; all our substance is embarked in the venture; yet the profession are to be the men (as a body so well remunerated) that are to be the only gratuitous contributors. In point of argument, I cannot understand this necessity.

Had the profession been accused of lowering its dignity by suffering the churchwarden to put up the diseases to the lowest lowest bidder, or the county magistrate for the prison—this was an attack not to be defended, for here, alas, interest operates, and all diseases not immediately affecting life are neglected, and those that do bring it into danger are scantily supplied. Better far would every parish fare to find its own medicine, and trust to the benevolence of the profession to supply a gratuitous attendance. Wherever a call of this kind occurs, there is always competition, and, no doubt, parish situations would be as respectably filled as other public institutions.

I trust the Medical and Surgical Worcestershire Society will not be found either offering an insult to the profession, or lessening their dignity; but that they merit thanks for their humble exertions for the bettering the best interests of the poor. As a member of that Society, I cordially signed the petition, and would again sign it, in spite of the cry of want of feeling.

*Worcestershire; August* 27, 1817.

---

*** We suspect that Humanitas, in his zeal for the profession, has overlooked the difference between town and country practice. In London a poor wretch is always within the sphere of a medical man. In the country he may be at the distance of several miles, and the parish-officer not feel himself at liberty to make the remuneration he may wish. There appears to us no more reason why medical men should give their time and labour for nothing, than that they should be charged higher for poor-rates. Every man's time is his money, or the most important part of his stock in trade. Let us compare the charges of country solicitors on these occasions with those of surgeons, and recollect the difference of time and labour.—EDIT.

